# Residual Stress Relief in Metallic Materials: Traditional Methods, Emerging Techniques, and Multi-Field Synergies

**DOI:** 10.3390/ma19122431

**Published:** 2026-06-06

**Authors:** Shushan Hu, Gang Huang

**Affiliations:** 1 Department of Mechanical and Traffic Engineering, Ordos Institute of Technology, Ordos 017000, China; 2 School of Energy Power and Mechanical Engineering, North China Electric Power University, Beijing 102206, China

**Keywords:** residual stress, mechanical properties, stress relief technologies, magnetic–vibration stress relief, multi-field coupling

## Abstract

Residual stress, an inevitable byproduct of manufacturing processes, significantly compromises the mechanical integrity, formability, and dimensional stability of metallic components. A comprehensive understanding of residual stress evolution and effective mitigation strategies is therefore critical for preventing structural failure. This review systematically examines the generation mechanisms, multi-scale classifications, and performance impacts of residual stresses in metallic structures. We critically evaluate the evolution of stress relief technologies, transitioning from traditional thermal and mechanical methods—which often suffer from high energy consumption, environmental concerns, or geometric distortion—to emerging non-thermal single-field techniques such as ultrasonic, magnetic, and electropulsing treatments. Crucially, this paper highlights a paradigm shift toward multi-field coupling strategies. By synergistically integrating thermal, magnetic, and vibrational energies, novel approaches like Combined Magnetic–Vibration (CMVSR), Thermal–Vibration (TVSR), and Thermal–Magnetic (TMSR) stress relief demonstrate superior stress relaxation efficacy while maintaining microstructural stability and minimizing energy expenditures. Ultimately, this review provides a comprehensive roadmap for selecting appropriate mitigation strategies and outlines the future trajectory of eco-friendly, high-efficiency stress relief in advanced manufacturing.

## 1. Introduction

Ferromagnetic materials, particularly steel, serve as the cornerstone of modern industrial manufacturing and infrastructure. Ferromagnetic materials possess strong inherent magnetism, capable of achieving magnetic saturation in relatively low magnetic fields (less than a few A/mm), and exhibit nonlinear magnetic permeability in response to field strength variations. Despite their utility, the processing of these materials—ranging from initial rolling and heat treatment to final machining—inevitably introduces complex residual stress fields. These stresses act as hidden defects that can severely compromise the structural integrity and functional performance of the final product.

**Figure 1:**
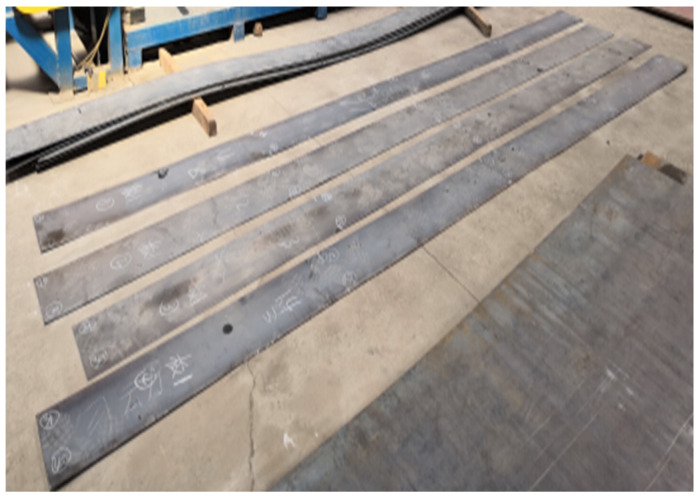
Side bending of a straight steel plate after slitting.

**Figure 2:**
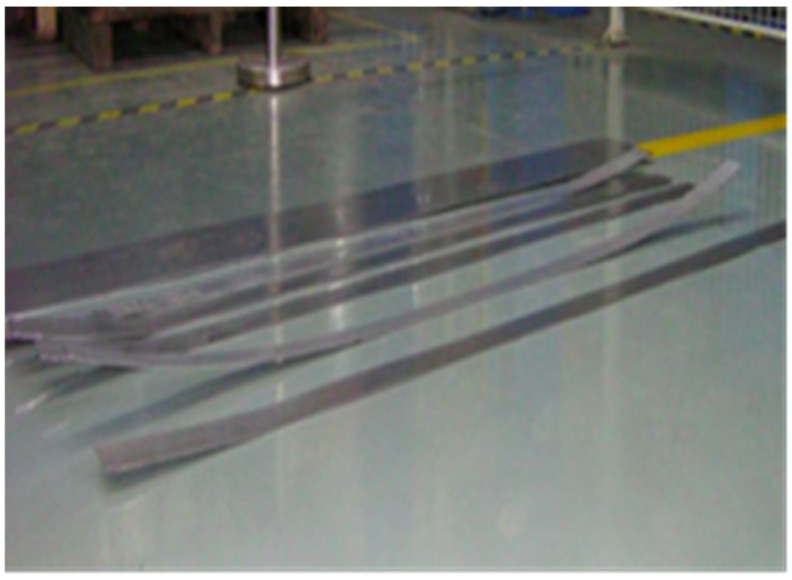
Warping of a flat steel plate after slitting.

Residual stress is generated throughout the entire manufacturing lifecycle. It affects both structural and functional materials during preparation processes (e.g., rolling, annealing, quenching) and deep processing stages. Recent numerical investigations on heavy-wall offshore pipelines have demonstrated that this deformation inhomogeneity across the wall thickness fundamentally dictates the final mechanical properties and service reliability of large-scale structures [1]. Although often macroscopically latent, residual stress acts as an intrinsic initial condition for subsequent fabrication. Its presence invariably compromises dimensional accuracy and can lead to unpredictable failure modes. As illustrated in Figure 1, a high-strength hot-rolled automobile frame steel plate, initially flat, exhibits significant side bending (camber) after slitting due to the release of internal stress. Similarly, Figure 2 demonstrates severe warping in a cold-rolled steel strip intended for home appliances following longitudinal cutting. These distortions—including warping, buckling, and side bending—are direct macro-mechanical manifestations of the inhomogeneous residual stress fields within the material.

From a thermodynamic perspective, materials containing residual stress are in a metastable, high-energy state. During subsequent processing or service, the material strives to minimize its total free energy. This re-equilibration involves the release of stored elastic strain energy, which drives plastic deformation or fracture [2]. Consequently, residual stress has become a critical quality index that must be rigorously controlled, particularly in advanced materials such as high-strength structural steel and high-permeability silicon steel. Given the dominance of ferromagnetic materials in global infrastructure, developing efficient, fast, and controllable stress reduction technologies remains a pressing challenge. While previous review articles have comprehensively covered classical thermal and mechanical stress relief methods, there is a critical gap regarding the systematic evaluation of emerging non-thermal techniques and their integration. Therefore, this article distinguishes itself by focusing on the transition from single-field treatments to advanced multi-field coupling technologies (e.g., combined magnetic–vibration and thermal-assisted coupling).

To ensure a rigorous analytical framework, a systematic literature search was conducted using databases including Web of Science, Scopus, and Engineering Village. Keywords such as “residual stress reduction,” “vibration stress relief,” “magnetic treatment,” and “multi-field coupling” were utilized. Emphasis was placed on peer-reviewed journal articles published primarily over the last two decades, with a specific focus on screening recent advancements (2018–2026) in numerical modeling and hybrid technologies to reflect the current state of knowledge. This review synthesizes these findings to evaluate existing methods critically and outline future directions in modern eco-friendly manufacturing.

## 2. The Concept and Source of Residual Stress

Throughout the entire material production lifecycle, from smelting to final usage, various heat treatment and mechanical processing methods are employed, including casting, rolling, welding, cutting, grinding, milling, and assembly. These processes inevitably generate varying levels of internal stress within the material. Residual stress is defined as the stress remaining in a material after all external loads and environmental influences have been removed. Generally, residual stress is categorized into two types: the initial stress field, which is generated during primary shaping processes, and the processing residual stress, which is introduced during subsequent mechanical processing. It is an inherent, self-equilibrating internal stress field that constitutes a fundamental property of the material [3]. The presence of residual stress poses significant challenges to material processing and utilization; consequently, research into controlling and minimizing these stresses has become a critical priority.

In 1860, Wöhler conducted pioneering fatigue research on railway axles, identifying that latent internal stresses—now formally recognized as residual stresses—significantly contributed to unpredictable structural fractures [4]. Macroscopically, residual stress is classified as either tensile or compressive based on its manifestation. Tensile residual stress occurs when the lattice or unit body acts to expand upon the removal of external loads, whereas compressive residual stress acts to contract. Based on the scale of observation, residual stress is fundamentally rooted in incompatible internal strains caused by non-uniform plastic deformation, and is divided into three categories. Macroscopic stress (Type I) extends over large distances and arises from bulk thermal gradients or uneven mechanical working. Mesoscopic stress (Type II) operates at the grain scale. It occurs because adjacent grains—due to differing crystallographic orientations and yield anisotropies—deform disproportionately under the same macroscopic load, generating intergranular mismatch stresses. Microscopic stress (Type III) exists at the sub-grain or atomic level, primarily originating from lattice defects such as dislocations and vacancies. Advanced models of screw dislocation interactions under steady-state thermal loading have further elucidated how these microscopic defects influence interfacial crack initiation and propagation [5]. Therefore, the complex residual stress profile is the superimposed result of these macroscopic gradients and microscopic inhomogeneities [6,7].

The origins of residual stress can be broadly categorized into extrinsic and intrinsic factors. Extrinsic factors include processing methods and environmental conditions, while intrinsic factors relate to structural variations within the material. Often, residual stress resulting from uneven deformation arises from a combination of both. For example, extrinsic machining operations (such as cutting, bending, rolling, or drawing) interact with intrinsic factors like structural and grain anisotropy to cause variations in yield behavior. Temperature fluctuations also induce residual stress. Due to the material’s complex structure and asymmetric geometry, the thermal conductivity varies across different regions, creating steep temperature gradients that generate macro-scale residual stresses. Conversely, micro-scale residual stresses arise intrinsically from anisotropy in the crystal structure and grains, which leads to localized differences in physical properties (e.g., thermal expansion coefficients) across the material matrix. Additionally, chemical changes within the material can also result in residual stress, which is often attributed to external causes.

**Figure 3:**
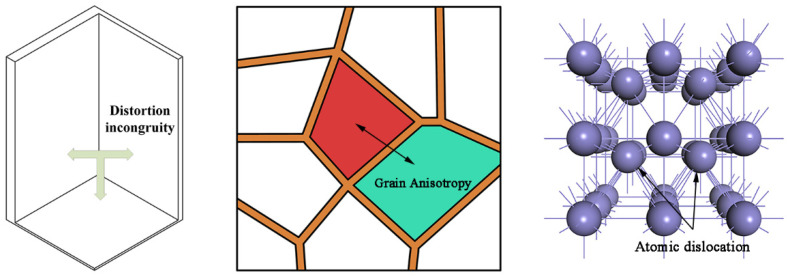
The reasons for the different scales of residual stress.

Fundamentally, residual stress is caused by a multitude of factors but is ultimately attributed to non-uniform plastic deformation [6]. Inhomogeneous deformation resulting from temperature changes or chemical reactions leads to an uneven distribution of strain. To maintain continuity, internal forces must be generated to balance the stressed and unstressed areas, as illustrated in Figure 3. The residual stress formed during this process is an inevitable outcome of various machining procedures. For example, quenching ferromagnetic materials creates a substantial residual stress field; subsequent material removal during machining disrupts this original equilibrium, resulting in deformation. The degree of deformation depends on factors such as the stiffness and symmetry of the ferromagnetic material. At the microscopic level, the stress distribution is highly intricate due to the presence of numerous dislocations, vacancies, grain boundaries, and sub-grain boundaries. The combined impact of these factors produces complex residual stress patterns within the material.

## 3. Effect of Residual Stress on Materials

**Figure 4:**
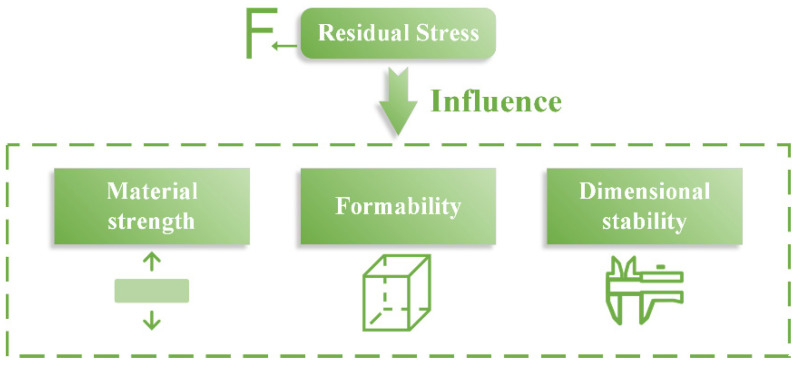
The effect of residual stress on material performance.

Generally, residual stress exerts both beneficial and deleterious effects on material performance, as illustrated in Figure 4. Therefore, a comprehensive understanding of how residual stress impacts material properties is critical for effectively utilizing its positive effects while mitigating its negative consequences.

### 3.1. Influence of Residual Stress on the Strength and Service Performance of Metallic Materials

In metallic materials, the residual stress state remains relatively stable during purely elastic deformation; significant alteration or generation of new residual stress typically occurs only during subsequent plastic deformation. This evolution is highly dependent on the material’s composition and yield behavior. Notably, the presence of compressive residual stress can enhance structural stability by effectively increasing the nominal yield stress, as the internal compressive field offsets applied tensile loads. Furthermore, the structural stiffness of assembled components is highly sensitive to the internal stress state at contact interfaces; for complex geometries like rotating dovetailed blades subjected to multiaxial stress, connection stiffness is fundamentally governed by the macro-micro interface topography and localized residual stress distributions [8].

While material hardness is predominantly governed by microstructure and phase composition (e.g., martensite fraction, precipitate density), residual stress also plays a measurable role. Tensile residual stress acts synergistically with applied indentation loads, facilitating the early onset of plastic flow and resulting in decreased apparent hardness. Conversely, compressive residual stress opposes the applied load, inhibiting localized plastic deformation and artificially elevating the measured hardness. Recent nanoindentation studies (e.g., in 2025) have quantitatively confirmed this mechanical synergy, demonstrating that both the apparent hardness and yield resistance increase linearly in proportion to the magnitude of the internal compressive stress [9].

Furthermore, residual stress exerts a profound influence on fatigue strength. For instance, surface compressive residual stress induced by cold rolling significantly increases the fatigue limit. Compressive stress generated through heat treatment is often even more effective in this regard. The redistribution of residual stress under alternating loads can further improve fatigue strength, provided the redistribution occurs in a favorable manner. Recent fatigue relaxation models and neutron diffraction experiments [10] emphasize that accurately quantifying this dynamic cyclic stress relaxation is critical, as the early-stage redistribution of compressive stress can significantly alter crack initiation sites and effectively extend the overall low-cycle fatigue life. Guo et al. [11] established that mild grinding generates significant surface compressive stress, which prolongs the fatigue life of components. Conversely, tensile residual stress can drastically reduce fatigue life, as identified by Tang et al. [12]. In heavy-duty equipment such as shearers, the reliability of key components is critically sensitive to such localized stress distributions induced during manufacturing [13]. Furthermore, recent advancements by Hills et al. [14] revealed significant local variations in residual stress at identical sub-surface depths, demonstrating that integrating advanced residual stress profiling with machine learning algorithms serves as a highly accurate predictor for component fatigue life.

Finally, residual stress plays a crucial role in crack initiation and propagation, particularly in brittle materials where tensile residual stress provides the driving force for crack formation. Crack propagation is often accelerated by stress concentration due to corrosion. Conversely, compressive residual stress can hinder Stress Corrosion Cracking (SCC). To optimize performance, a specific level of compressive residual stress is generally preferred to enhance strength, whereas tensile residual stress is undesirable as it compromises material integrity.

### 3.2. Influence of Residual Stress on Machining and Forming Properties

With the advancement of manufacturing technologies, sectors such as 3D printing, thick coating, laminated plating, and high-strength steel production have become increasingly prominent. Consequently, the influence of residual stress on processing and forming has gained significance. Among these, Additive Manufacturing (AM), or 3D printing, has garnered considerable attention for its ability to produce complex shapes on demand [15]. AM employs liquid, semi-solid, or powder feedstocks to print components directly to near-net or final shape [16]. As this technology matures, its role in industrial manufacturing is expected to expand. For instance, in the Selective Laser Melting (SLM) of individualized femur implants, precise process optimization is mandatory to mitigate the high residual stresses that otherwise compromise the implant’s biocompatibility and fatigue resistance [17].

However, Li et al. [18] demonstrated that AM inevitably generates residual stress due to repeated heating and cooling cycles, leading to material deformation. This unique thermal cycle involves the simultaneous melting of new layers and the reheating of previously solidified layers, inducing complex stress fields. Mercelis et al. [19] explained that the high-energy heat source causes rapid expansion, followed by shrinkage during cooling; the constraint provided by the underlying layers results in compressive stress. Lu et al. [20] identified scanning speed, laser power, and build direction as primary factors influencing AM residual stress. They found that a 2 × 2 mm^2^ island scanning strategy effectively reduced global residual stress; however, it increased susceptibility to surface cracking. Mechanistically, this occurs because the borders of the small scanning islands create localized thermal overlaps and steep temperature gradients, leading to concentrated tensile stress peaks at the melt pool boundaries that initiate micro-cracks. Kruth et al. [21] investigated various scanning paths, revealing that deformation is closely linked to layer deposition patterns (e.g., checkerboard strategies), with different strategies yielding distinct stress profiles.

In the steel strip industry, flatness is a vital quality parameter. Significant uneven deformation during rolling or heat treatment leads to flatness defects, primarily curling and warping. Residual stress is the mechanical driving force behind these defects. Non-uniform stress distribution causes issues such as warpage [22], curling, and camber (side bending). Low-stiffness materials are particularly susceptible to deformation caused by uneven residual stress. In the high-precision machining of thin-walled boxes, geometric errors often arise from stress-induced distortion, necessitating advanced error gradient compensation methods to ensure structural accuracy [23].

Residual stress is dynamic; its distribution evolves following different processing steps. Material removal during machining disrupts the original stress equilibrium. If a significant volume of material is removed, the remaining material must deform to achieve a new state of equilibrium, resulting in machining distortion.

### 3.3. Influence of Residual Stress on Dimensional Stability

Dimensional stability is defined as a material’s ability to resist permanent deformation and maintain its geometry over time in the absence of external loads. Long-term dimensional instability arises from several factors: thermodynamic instability of the phase or structure, stress relaxation during storage or assembly, and material anisotropy [24].

Residual stress is a dominant factor governing dimensional stability [25]. Xu et al. [26] investigated the efficacy of thermal vibration treatment on aluminum alloys and composites, finding it beneficial for stabilization. Tang et al. [27] demonstrated that cryogenic thermal cycling treatment effectively mitigates residual stress while promoting the formation of nanoscale precipitates and stabilizing dislocation networks. These microstructural modifications synergistically enhance the material’s yield strength and its resistance to microscopic plastic deformation, thereby ensuring long-term dimensional stability. Qu et al. [28] emphasized that SiCp/Al composites require high dimensional stability, noting that the residual stress in the as-cast composite significantly impacts its long-term stability under thermal cycling conditions.

### 3.4. Measurement Techniques for Residual Stress

The accurate quantification of residual stress is crucial for validating the efficacy of any reduction technology [29]. Measurement techniques are broadly classified into destructive, semi-destructive, and non-destructive methods. Non-destructive diffraction techniques are the most prevalent. X-ray Diffraction (XRD) measures the change in atomic lattice spacing (nearest-neighbor distances) near the surface (typically within 10–30 μm depth) to calculate strain and stress based on Bragg’s Law [30]. For deeper penetration, Neutron Diffraction can map 3D residual stress fields within bulk materials (up to several centimeters deep in steel), making it ideal for evaluating complex macroscopic stresses (Type I) [31]. Alternatively, the Hole-Drilling Strain Gage Method (a semi-destructive technique guided by ASTM E837 [32]) is widely used in engineering due to its portability and reliability; it determines the stress profile by incrementally drilling a small hole and measuring the resulting relaxed strains via a surface strain rosette [33].

## 4. Residual Stress Reduction Methods and Technologies

Residual stress generation is an inevitable byproduct of manufacturing, often leading to severe structural degradation. Consequently, mitigating residual stress to induce relaxation is a critical processing step, often considered the ultimate stage of material fabrication. This article categorizes existing reduction technologies into traditional and emerging approaches, providing a comprehensive and critical analysis of each.

### 4.1. Traditional Residual Stress Relief Methods and Techniques

#### 4.1.1. Natural Treatment

**Figure 5:**
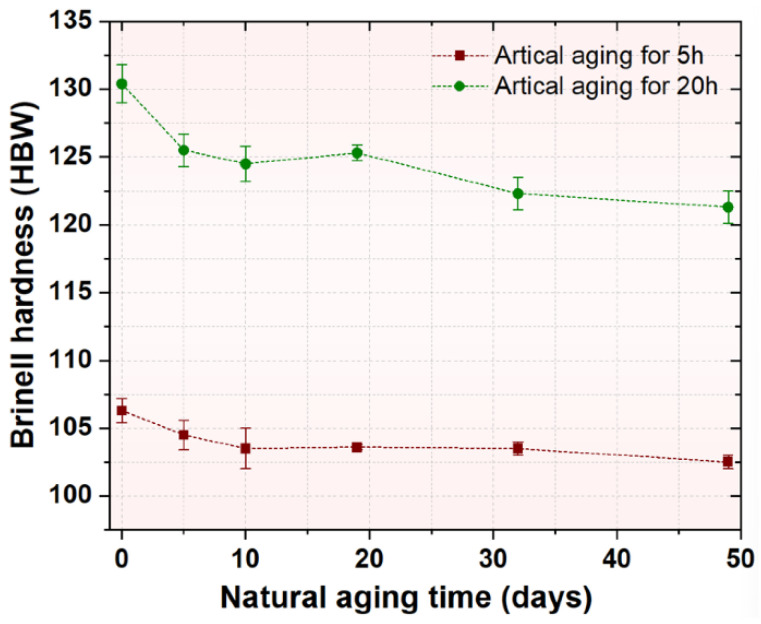
Brinell hardness vs. natural aging time for T6 heat-treated (under- and peak-aged) 2024 alloys [36].

Natural treatment, historically referred to as “natural seasoning,” is the most primitive approach to stress relief. It involves exposing workpieces to the natural environment for extended periods, where ambient temperature fluctuations induce repeated thermal expansion and contraction. Recent microstructural studies by Chen et al. [34] demonstrate that this prolonged exposure facilitates localized micro-creep and the evolution of unstable precipitates in regions of high stress concentration, as shown in Figure 5, thereby enhancing dimensional stability over time. While it offers the advantage of zero energy consumption, recent industrial reviews [35] indicate that its application has been largely phased out in modern agile manufacturing. The excessively long treatment duration (months to years), substantial space requirements, and uncontrollable environmental boundary conditions render it incompatible with contemporary high-efficiency production standards.

Summary of Achievements and Deficiencies: Natural aging leverages ambient thermal fluctuations to facilitate localized micro-creep and the stabilization of precipitates without external energy input. However, its critical drawbacks include an excessively long processing cycle (months to years), substantial space requirements, and uncontrollable environmental boundary conditions, rendering it largely incompatible with modern agile manufacturing standards.

#### 4.1.2. Thermal Treatment

**Figure 6:**
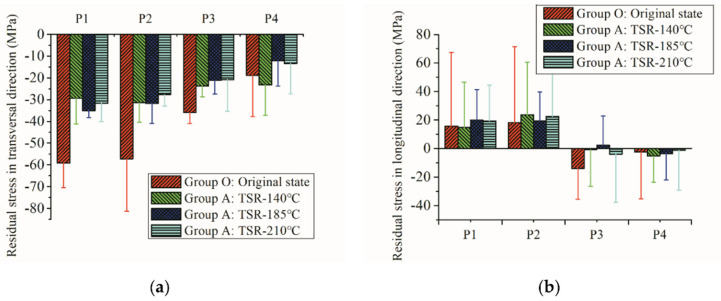
Residual stresses in the transversal direction (**a**) and longitudinal direction (**b**) at each measurement point, before and after thermal stress relief (TSR) treatment at different temperatures (each bar represents the average values along the depth direction, and the one with the largest amplitude among the six measured values is expressed by the error bars.) [39].

Thermal treatment, conventionally known as stress relief annealing, is a widely adopted method for mitigating residual stress [37], as illustrated in Figure 6. The process entails gradually heating the material from room temperature to a plateau slightly below the recrystallization temperature, maintaining this temperature for a specific duration (holding time), and subsequently subjecting the material to slow cooling—typically furnace cooling.

Precise control over the process parameters is imperative. Governed by the fundamental Arrhenius equation for thermal activation, holding times and peak temperatures (typically 500–700 °C for structural steels) must be strictly calibrated. Furthermore, modern FEM heat-transfer models are now frequently employed to optimize these parameters, reducing the margin of error in stress prediction to below 5%, compared to the >15% margin of error common in traditional empirical furnace settings. Excessive temperatures or prolonged holding times can lead to microstructural degradation, such as graphitization (in cast irons) or grain coarsening, which ultimately compromises mechanical strength. Furthermore, rapid heating rates—particularly in thin-walled components—can induce severe thermal stress; if this stress exceeds the material’s yield limit, it may result in cracking or distortion. Similarly, improper cooling rates can hinder the stress relief effect, potentially reintroducing residual thermal stress that negates the benefits of the treatment. While optimized thermal treatment can effectively reduce macroscopic residual stress by 40–80% in carbon and low-alloy steels (depending on peak temperature, hold time, and initial stress magnitude), it may not fully eliminate microscopic (Type II and III) residual stresses pinned by stable defects. Additionally, the method is associated with high energy consumption, significant operational costs, and environmental concerns. Despite these limitations, it remains a dominant technique in mechanical manufacturing due to its ability to significantly stabilize workpiece dimensions and lower global residual stress levels. Falodun et al. [38] reported that increasing the heat treatment temperature effectively reduces residual stress, thereby decreasing susceptibility to stress corrosion cracking (SCC) and lowering the electrochemical activity of welded samples.

Pan et al. [40] demonstrated that thermal treatment is highly effective for steel structures, provided that heating and cooling rates are strictly controlled. It is generally accepted that the magnitude of stress reduction is influenced more by the treatment temperature than by the holding time. Huang et al. [41] investigated the impact of thermal treatment on microstructure, finding that the method facilitates grain refinement, improves ductility, and enhances resistance to corrosion cracking. Liu et al. [42] established a theoretical foundation for optimizing thermal treatment processes by elucidating the evolution of residual stress in welded structures. Xiao et al. [43,44] reported that thermal treatment not only reduces residual stress and improves yield strength but also effectively eliminates voids in copper matrices, leading to reduced dislocation density in sub-grains and more uniform grain orientation. Wu et al. [45] developed a finite element simulation model to predict stress relief during thermal treatment and validated it experimentally, offering new methodologies for process optimization. Furthermore, thermal treatments applied to additively manufactured components can effectively reduce macroscopic stresses, though localized micro-scale residual stresses often persist due to complex thermal histories [46]. Finally, Fogel et al. [47] employed laser-induced thermal treatment on guide rails, discovering that the process resulted in a threefold decrease in electrical resistivity and induced grain growth, leading to the formation of a stabilized microstructure.

Summary of Achievements and Deficiencies: Thermal annealing is the most mature industrial technology, capable of reducing macroscopic stresses by up to 80% while simultaneously refining grains and improving ductility. Nevertheless, it is fundamentally limited by high energy consumption, environmental pollution, and the risk of microstructural degradation (e.g., grain coarsening). For complex or thin-walled components, improper heating/cooling rates can also trigger severe secondary thermal stresses and macroscopic distortion. This risk is particularly critical under extreme thermal shock conditions, where transient dynamics in materials with geometric discontinuities (e.g., cutouts) can rapidly convert thermal gradients into destructive internal stresses, leading to immediate structural failure [48].

#### 4.1.3. Mechanical Tensioning

Mechanical tensioning involves applying a macroscopic tensile load to a component to induce controlled plastic deformation. When the superposition of the applied tensile stress and the pre-existing residual stress exceeds the material’s yield strength, localized plastic flow occurs, thereby redistributing and relaxing the internal stress field. The fundamental efficacy of this approach was comprehensively established in the authoritative work by Richards et al. [49], who demonstrated that Global Mechanical Tensioning (GMT) significantly mitigates peak longitudinal residual stresses in welded alloys. At the microstructural level, recent research by Zhu et al. [50] confirmed that applying specific tensile deformation (e.g., 3%) provides sufficient activation energy to mobilize pinned dislocations at grain boundaries, leading to uniform dislocation annihilation and significant macroscopic stress relaxation.

**Figure 7:**
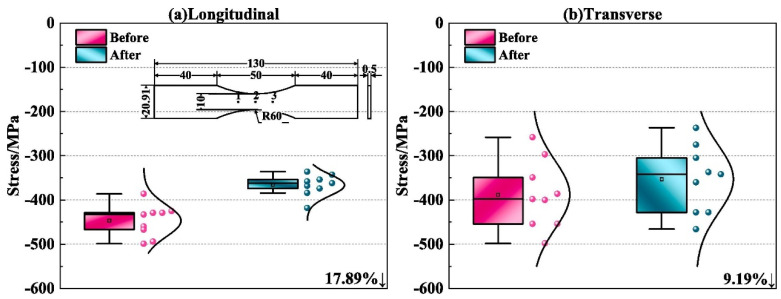
Residual stress changes before and after fatigue treatment: (**a**) longitudinal; (**b**) transverse [51].

While static tensioning is highly effective, recent advancements have expanded this concept into dynamic and multi-field domains to address complex geometries. As demonstrated by Huang et al. [51], the application of fatigue alternating loading—a cyclic form of mechanical stretching—serves as a potent mechanism to homogenize stress peaks via micro-plastic flow without causing the macroscopic buckling associated with severe static overloads, significantly altering both longitudinal and transverse residual stresses (Figure 7). Furthermore, Ilman et al. [52] introduced in situ rolling tensioning during friction stir welding, proving that synchronous mechanical stretching effectively diminishes welding distortion and significantly improves fatigue performance. For structures where pure mechanical stretching is geometrically prohibitive, Thermo-Mechanical Tensioning (TMT) has emerged as a viable alternative. Bora et al. [53] successfully applied TMT to orthotropic steel decks, utilizing localized heating coupled with equivalent mechanical loading to induce precise elastic-plastic stress relaxation.

Summary of Achievements and Deficiencies: Mechanical tensioning effectively induces localized plastic flow to redistribute internal stress fields, achieving high relaxation efficiency in simple geometries. The primary deficiency of this single-field approach is its extreme dependency on structural geometry. For components with complex cross-sections or non-uniform stiffness, applying macroscopic tensile loads entails high risks of irreversible macroscopic distortion, localized necking, or structural failure.

#### 4.1.4. Impact and Shockwave Treatments (Hammering and Explosion)

Impact-based methods rely on the rapid transfer of high-strain-rate mechanical energy to induce severe plastic deformation, compensating for internal shrinkage strains and redistributing residual stresses. These techniques are classified by their energy scale and penetration depth.

**Figure 8:**
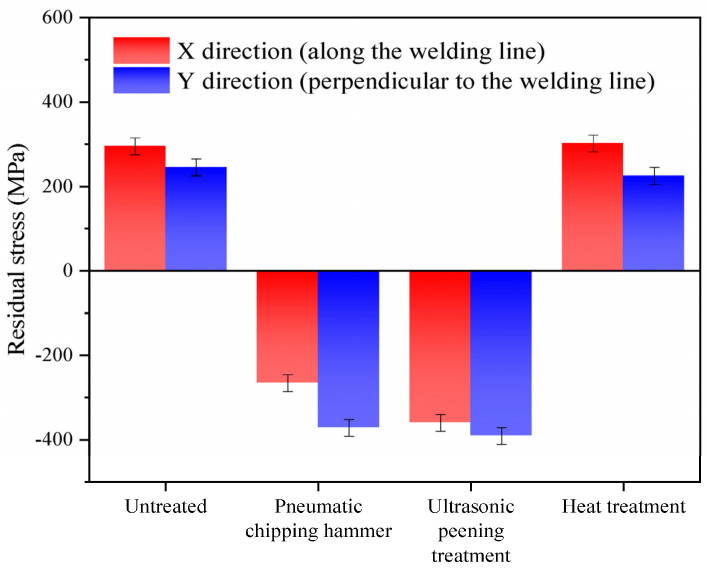
Residual stress of welded joints with different treatments [56].

Hammer Peening: At the localized surface level, hammer peening applies mechanical impacts to specific areas, such as weld toes. While empirically used for decades, modern computational materials engineering has revitalized this method. Recent Finite Element Method (FEM) simulations by Zha et al. [54] and Hu et al. [55] have demonstrated that peening impacts induce a highly complex multiaxial stress state. Their layer-by-layer continuous coupled simulations accurately predict the conversion of deleterious tensile residual stresses into beneficial compressive stress layers up to specific depths (Figure 8). These numerical models confirm that the induced plastic flow significantly alters local yield criteria, robustly retarding crack growth in dissimilar steel joints.

Explosion Treatment (ESR): Scaling up from localized surface impacts to the macroscopic bulk level, Explosive Stress Relief (ESR) historically utilized high-velocity shockwaves generated by detonating explosives to relax peak stresses in heavy-duty components. While recent investigations into extreme blast loading by Li et al. [57] unequivocally demonstrate the immense energy transfer and deep penetration capabilities of such shockwaves in massive steel infrastructure, the intentional use of chemical explosives for stress relief is now considered largely obsolete. Due to extreme safety hazards, acoustic pollution, and uncontrollable macro-distortion, ESR has been practically phased out of modern precision manufacturing, relegated strictly to highly specialized, legacy outdoor engineering applications.

Summary of Achievements and Deficiencies: Impact-based methods, particularly high-strain-rate shockwaves, can effectively convert deleterious tensile stresses into beneficial compressive layers. However, Explosive Stress Relief (ESR) is practically obsolete in precision manufacturing due to extreme safety hazards, acoustic pollution, and uncontrollable deformation. Similarly, hammer peening is restricted by surface damage risks and the potential for secondary stress concentration at the impact boundaries.

#### 4.1.5. Vibration Treatment

**Figure 9:**
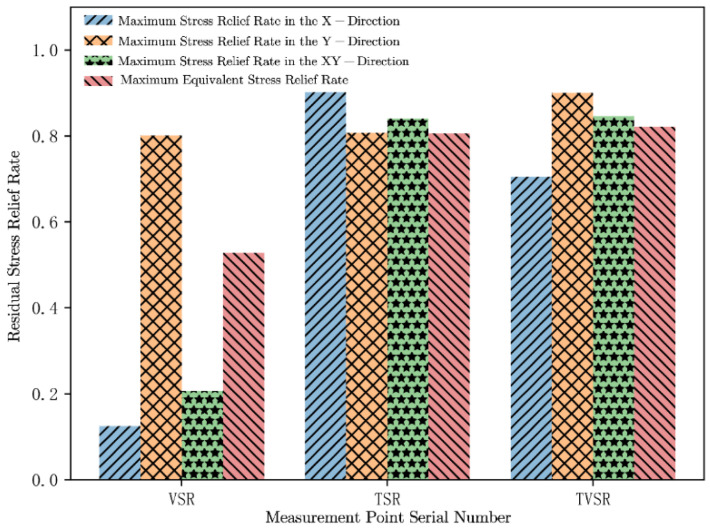
Residual stress homogenization rate of 2219 aluminum alloy ring after various stress relief treatments [59]. Here, the specific aims and parameter categories are distinct: VSR (Vibratory Stress Relief) relies on dynamic mechanical parameters (e.g., 20–100 Hz resonance) aimed strictly at overcoming local lattice friction to induce micro-yielding; TSR (Thermal Stress Relief) utilizes thermodynamic parameters (e.g., peak temperatures of 150–250 °C) aimed at accelerating atomic diffusion and macroscopic stress relaxation; TVSR (Thermal–Vibration Stress Relief) couples both fields, aiming to utilize thermal activation to lower the mechanical yield threshold, thereby achieving maximum stress homogenization with minimal input energy.

Vibration Stress Relief (VSR), developed industrially during the 1950s, utilizes mechanical resonance to reduce residual stress, as illustrated in Figure 9. Due to its high efficacy and operational simplicity, VSR has been widely adopted in manufacturing. A distinct advantage of VSR over traditional thermal treatment is that it is not constrained by furnace dimensions. This characteristic makes it particularly adaptable for large-scale and complex structural components where maintaining dimensional stability is paramount [58].

The theoretical foundation of VSR traces back to the early 1900s, with concepts initially proposed by the physicist Stratt, who established the underlying principles. Research on VSR process parameters and residual stress characterization in aluminum alloys has provided important experimental and simulation support for the method [60,61]. By the 1970s, Wozney [62] formalized the mechanism, establishing the fundamental stress superposition formula: *σ_d_* + *σ_r_* ≥ *σ_s_*, where *σ_d_* is the applied dynamic stress, *σ_r_* is the internal residual stress, and *σ_s_* is the material’s yield limit. To achieve effective relaxation without structural damage, working parameters must be strictly controlled within specific ranges: the excitation frequency is typically locked at 95%–100% of the component’s natural resonant frequency, with an acceleration range of 20–50 m/s^2^ and a treatment duration of 15–45 min. Parameter ranges to be strictly avoided include prolonged resonance dwell times (>60 min), which risk high-cycle fatigue accumulation. Furthermore, determining these parameters has transitioned from traditional experimental trial-and-error approaches to advanced Computer-Aided Design (CAD) and FEM techniques. While empirical experimental methods rely on surface strain gauges with a typical margin of error of approximately ±10–15%, modern CAD/FEM modal analyses can accurately predict optimal excitation locations and frequencies, reducing the operational margin of error to below 5%. In fact, the precise implementation of Vibratory Stress Relief (VSR) relies heavily on understanding the inherent dynamic characteristics of target components. Recently, sophisticated numerical and analytical modeling methods have been developed to investigate the free and forced vibration behaviors of complex engineering structures, ranging from pre-twisted rotating blades and asymmetric herringbone gears to heavy machinery platforms, multiscale composite plates, and coupled-field annular structures [63,64,65,66,67,68]. In addition, advanced fatigue life prediction and dynamic response analyses for cyclically loaded industrial systems have further highlighted the importance of structural geometry, modal behavior, and stress redistribution characteristics in vibration-assisted stress relief processes. Incorporating these structural dynamic analyses and fatigue evaluation models provides a more robust theoretical basis for optimizing VSR excitation parameters and mitigating high-cycle fatigue damage in geometrically complex industrial components. For instance, in their comprehensive evaluation of stress relief methodologies, Gao et al. [69] systematically investigated the pure vibratory stress relief (VSR) process on 7075 aluminum alloys. Using both experimental and simulation approaches, their baseline VSR studies demonstrated that applying optimized dynamic vibration parameters alone can induce significant micro-yielding, thereby effectively reducing and homogenizing the machining-induced residual stresses.

In the 21st century, VSR technology has advanced significantly, driven by improvements in sensing and measurement capabilities. Gao et al. [70] employed finite element analysis combined with Charpy impact testing to study the effect of VSR on dissimilar steel (Q345/316L) welded joints, demonstrating that VSR improves impact toughness by approximately 26% and effectively reduces welding residual stress through dislocation-driven microplastic deformation. Gong et al. [71] applied VSR to complex thin-walled aviation components, reporting that the treatment not only reduced residual stress but also homogenized its distribution, thereby enhancing dimensional stability and shape retention. Ebrahimi et al. [72] demonstrated that optimizing the vibration frequency to approximately 95% of the natural frequency (sub-resonant zone) maximizes stress reduction efficiency. Kaçar et al. [73] noted slight improvements in tensile strength, surface hardness, and fatigue life following treatment.

From a microstructural perspective, Lai et al. [74] posited that the dynamic stress induces lattice motion—a form of internal friction—where frequency and amplitude are critical factors; they suggested that variations in internal friction could serve as an indicator of fatigue evolution. Finally, Gao et al. [75] investigated the amplitude dependency, finding that while low-amplitude VSR positively impacts the fatigue life of 7075-T651 aluminum alloy, high-amplitude VSR can have deleterious effects.

Summary of Achievements and Deficiencies: Vibration Stress Relief (VSR) offers high efficiency and portability, particularly for large-scale structures unconstrained by furnace dimensions. Its limitations, however, lie in its sensitivity to resonant frequencies and mode shapes; high-stiffness or ultra-small components are often difficult to excite effectively. Furthermore, improper amplitude control poses a significant risk of initiating fatigue cracks at micro-defects, potentially compromising long-term structural integrity.

### 4.2. New Residual Stress Reduction Method and Technology

#### 4.2.1. Magnetic Treatment

**Figure 10:**
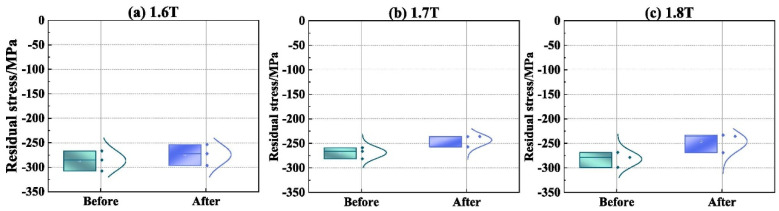
The relationship between the residual stress reduction ratio and the intensity of the magnetic treatment [77].

Magnetic treatment uses a dynamic, alternating magnetic field to optimize internal stress distribution in ferromagnetic materials. Early investigations demonstrated that magnetic treatment can reduce residual stress via the magnetoplastic effect, promoting dislocation mobility and microstructural homogenization [76], as illustrated in Figure 10. This method utilizes a dynamic, alternating magnetic field to optimize the internal stress distribution within ferromagnetic materials. The energy input is fundamentally governed by the magnetic field intensity and frequency, with optimal technical parameters strictly bounded within 0.5–2.0 T and 10–50 Hz for most structural steels. Modern CAD-integrated electromagnetic simulations are increasingly utilized to ensure uniform magnetic flux density across complex geometries, effectively reducing the operational margin of error to less than 5% compared to conventional empirical setups. Compared to traditional thermal annealing, magnetic treatment offers distinct advantages, including operational simplicity, low cost, minimal energy consumption, and environmental cleanliness. However, its primary limitation is that its effectiveness is largely restricted to ferromagnetic materials, limiting its applicability for non-magnetic alloys.

Continuous research has deepened the theoretical understanding of magnetic treatment. Early fundamental work by Al’shits et al. [78] observed that dislocations in NaCl crystals could slip under the influence of an external magnetic field, establishing a physical basis for magneto-plasticity. Bose investigated the effect of saturation magnetic fields on low-carbon steel, noting improvements in fatigue performance. Rong et al. [79] suggested that magnetic treatment is particularly effective in reducing tensile stress associated with welding deformation.

In terms of mechanical properties, Choi et al. [80] reported that while the elastic modulus decreased slightly, the yield strength, tensile strength, elongation, and fatigue life of the materials were improved following magnetic treatment. Wang et al. [81,82,83] demonstrated that magnetic treatment effectively homogenizes residual stress distribution, increases microhardness, and enhances fatigue life. Supported by recent XRD and TEM characterizations, they observed a concomitant increase in dislocation density and a transformation of magnetic domains from a labyrinth structure to a layered structure, suggesting that the primary mechanism is the enhancement of dislocation motion, which alleviates localized stress concentrations.

Further exploring the driving forces, Yan et al. [84] demonstrated that the Lorentz force induced by the magnetic field can promote dislocation slip, resulting in localized plastic deformation. They also posited that the Joule heat generated during the process contributes thermally to stress relaxation. Song et al. [85] elucidated the mechanism by scrutinizing changes in microstructure and magnetic domains. They postulated that magneto-induced deformation is predominantly the outcome of dislocation redistribution, which renders the deformation field more uniform and facilitates stress relaxation. Consequently, magnetic treatment modifies not only the internal stress state but also the surface morphology through its influence on magnetic domain structures.

Summary of Achievements and Deficiencies: Magnetic treatment provides a non-contact, energy-efficient, and eco-friendly solution by utilizing magneto-plasticity to promote dislocation motion and domain wall rearrangement. Crucially, its application is exclusively restricted to ferromagnetic materials. For non-magnetic alloys such as aluminum, titanium, or austenitic stainless steels common in aerospace applications, single-field magnetic intervention remains largely ineffective.

#### 4.2.2. Pulse Treatment

**Figure 11:**
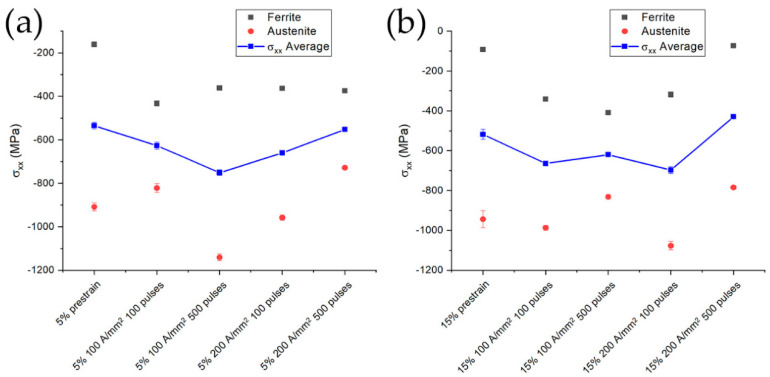
Evolution of transverse residual stresses in austenite (red circles) and ferrite (black squares) and their average (blue thick line) for specimens prestrained and electropulsed at (**a**) 5% and (**b**) 15% [87].

Pulse treatment, specifically referred to as Electropulsing Treatment (EPT) [86], is a high-energy method that modulates material properties by intermittently injecting transient electrical energy, as illustrated in Figure 11. The technique entails applying high-density current pulses to the conductive material. The interaction between the drift electrons and the crystal lattice induces electromigration and the electroplastic effect, which facilitates dislocation mobility and plastic deformation, thereby achieving residual stress reduction. EPT offers salient advantages, including rapid processing speed, high energy efficiency, operational simplicity, and a clean, pollution-free process.

The advancement of EPT technology has spurred a surge in sophisticated research, particularly captured in recent 2024–2025 reviews [88]. Current consensus elucidates that the EPT stress relief mechanism is fundamentally driven by a combination of thermal-activated energy coupling and the athermal electron wind force, which dramatically lowers the material’s flow stress under multiaxial loading conditions. Recent parametric investigations on aerospace alloys by Aprilia et al. [89] confirmed via high-resolution TEM characterizations that under high-density pulsed currents (10^3^–10^4^ A/mm^2^), the atomic nearest-neighbor distances at the dislocation core are locally relaxed without causing macroscopic thermal distortion. This phenomenon effectively alleviates internal strain gradients while maintaining dynamic stability within the crystal structure. Liang et al. [90] found that EPT effectively mitigates residual stress in high-speed steel cutting tools, thereby enhancing wear resistance and extending service life. Similarly, Mehdi et al. [91] employed EPT to treat Ti-6Al-4V alloy, reporting a significant decrease in tensile residual stress compared to untreated samples. Additionally, Yang et al. [92] observed that current pulses induced grain refinement in the fusion zone (FZ) and notably increased the ductility of Ti-6Al-4V Tungsten Inert Gas (TIG) welds.

Active research confirms that EPT possesses a unique capability to enhance material performance. Lobanov et al. [93] constructed a current pulse calculation model based on Maxwell’s equations, verifying it experimentally; their results indicate that EPT positively impacts metal structure, significantly reduces residual stress in welded joints, improves fatigue resistance and fracture toughness, and minimizes deformation in thin-walled components.

However, the efficacy of EPT can depend on coupling effects. Cai et al. [94] investigated the combination of pulsed magnetic fields and pulsed currents. Their experimental results indicated that, in their specific setup, pulsed magnetic field treatment alone reduced stress by approximately 10%, whereas pulsed current treatment alone actually increased stress by 20% (likely due to thermal shock). Crucially, the combination of both treatments yielded a stress reduction of about 60%. It is conjectured that the magnetic field facilitates dislocation detachment from pinning sites, while the pulsed current accelerates dislocation motion, demonstrating the power of multi-field coupling.

Summary of Achievements and Deficiencies: Electropulsing Treatment (EPT) utilizes ultra-high energy density to dramatically lower flow stress and facilitate dislocation mobility within milliseconds. Despite its speed and efficiency, EPT is limited to conductive materials. Moreover, the high current densities required can induce severe “current crowding” at internal micro-defects, leading to localized thermal shock, instantaneous melting, or electrical arc damage.

#### 4.2.3. Ultrasonic Impact Treatment

**Figure 12:**
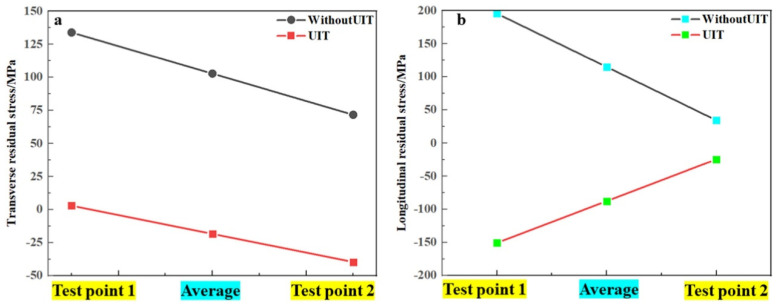
Residual stress distribution of Ni-based alloy overlayer before and after ultrasonic impact treatment: (**a**) transverse residual stress; (**b**) longitudinal residual stress [96].

Ultrasonic Impact Treatment (UIT), also known as ultrasonic peening, employs high-power ultrasonic transducers to subject the material surface to mechanical impacts at ultrasonic frequencies (typically >20 kHz). This high-strain-rate deformation generates a layer of compressive residual stress that modifies the original stress field. As illustrated in Figure 12, UIT has been demonstrated to significantly enhance the properties of the heat-affected zone (HAZ). Specifically, applying UIT to the weld toe of a component can improve the weld geometry, reduce surface defects, and substantially enhance fatigue strength.

A key benefit of UIT is its ability to produce dimensionally accurate components with minimal distortion. Compared to natural aging, the UIT process exhibits a significantly shorter production cycle and higher efficiency. Relative to thermal treatment, UIT is characterized by its equipment simplicity, low energy consumption, and lack of environmental pollution. Regarding welding residual stress, this method effectively reduces tensile stress near the weld and refines surface grains. However, it should be noted that the compressive stress layer induced by UIT is typically shallow, restricted to a limited depth from the surface [95].

Regarding the underlying mechanism, several studies have investigated the impact of UIT on stress reduction and material property enhancement. Yang et al. [97] reported that the acoustic energy absorbed by the grains during treatment directly influences the efficacy of residual stress reduction. Xiu et al. [98] established a finite element model (ABAQUS) simulating a vacuum vessel weld to investigate parameters such as the number of impact pins, impact strategy, pin diameter, and frequency; their results revealed a significant reduction in both longitudinal and transverse residual stresses in the weld and adjacent areas. Additionally, Liu et al. [99] investigated the impact of UIT on aluminum alloys, analyzing its dual influence on microstructure and macroscopic properties. Finally, Shalvandi et al. [100] studied the effect of UIT on 316 stainless steel. Their findings indicate that while extending the treatment duration improves stress reduction, it may weaken the material’s tensile properties. They also noted that in their specific study, the grain size was only weakly affected, suggesting that the primary mechanism was the influence on dislocation movement within the grains rather than significant grain refinement.

Summary of Achievements and Deficiencies: UIT is highly effective for enhancing the fatigue life of weld toes by inducing a deep nanocrystalline layer and high surface compressive stresses. Its main limitation is the shallow penetration depth; the beneficial compressive stress is typically confined to the subsurface (a few millimeters). For bulk residual stresses buried deep within thick-walled components, the rapid attenuation of ultrasonic energy renders this method insufficient.

#### 4.2.4. Cryogenic Treatment

**Figure 13:**
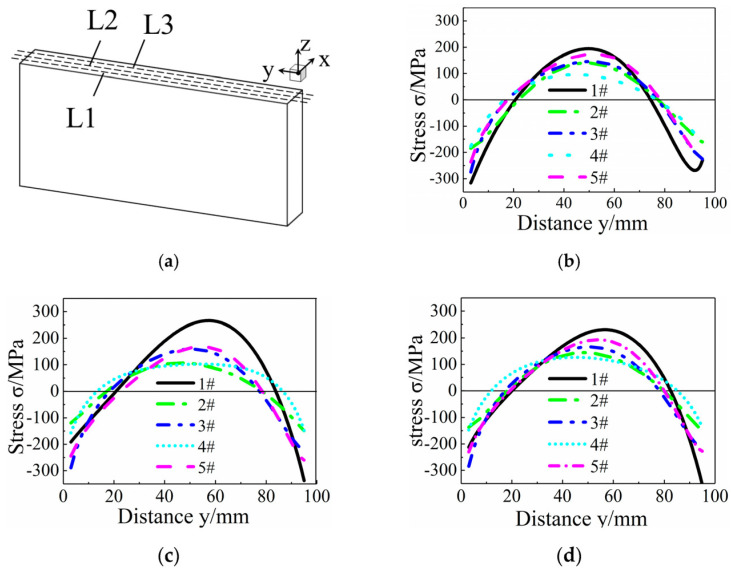
Comparison of stress along evaluation lines on cutting surface. (**a**) Schematic diagram of evaluation line. (**b**) Stress along line L1. (**c**) Stress along line L2. (**d**) Stress along line L3 [101].

Cryogenic treatment, often referred to as ultra-low temperature treatment or Deep Cryogenic Treatment (DCT), employs liquid nitrogen as a cooling medium to reduce residual stress by exposing materials to temperatures typically below −100 °C. As shown in Figure 13, cryogenic treatment significantly alters the residual stress distribution on the machined surface. The stress profiles measured along different evaluation lines (L1–L3) indicate that the treatment effectively homogenizes the stress field and reduces local stress concentration. This stress redistribution contributes to improved dimensional stability and mechanical performance of the processed component. Despite its effectiveness, cryogenic treatment is associated with relatively high operational costs and requires precise control of cooling and holding parameters to avoid excessive thermal gradients and potential cryogenic cracking [101].

Research has demonstrated the multifaceted benefits of this method. Li et al. [102] reported that cryogenic treatment effectively reduces residual stress while simultaneously increasing the volume fraction of precipitates. These microstructural modifications contribute to enhancements in strength, elongation, impact toughness, and wear resistance. In the context of WC-Co cemented carbides, Yong et al. [103] found that cryogenic treatment leads to improved hardness, compressive strength, wear resistance, and fatigue resistance. Although bending strength and toughness showed no significant variation, the study noted that magnetic saturation decreased with increasing coercivity. These mechanical improvements are attributed to stress relief and the martensitic transformation of the cobalt binder phase from face-centered cubic (σ-Co) to hexagonal close-packed (ε-Co). Furthermore, Araghchi et al. [104] reported substantial efficacy, achieving residual stress reductions of up to 71% alongside significant improvements in ultimate tensile strength. Additionally, Hariharan et al. [105] demonstrated that cryogenic treatment enhances the hardness and wear resistance of AISI D7 tool steel by driving the transformation of retained austenite into martensite.

Summary of Achievements and Deficiencies: Deep Cryogenic Treatment (DCT) excels in stabilizing dimensional accuracy and enhancing wear resistance by driving martensitic transformation and carbide precipitation. However, high operational costs and the consumption of liquid nitrogen limit its large-scale industrial adoption. Additionally, the steep temperature gradients during cooling can trigger “cryogenic cracking” or reintroduce secondary thermal stresses in brittle materials.

### 4.3. Analysis and Discussion on Methods and Techniques of Residual Stress Reduction

The reduction in residual stress is fundamentally a thermodynamic process involving energy dissipation and stress redistribution, which necessitates the input of external energy. When external energy is superimposed onto the material’s existing high-energy state (the residual stress field), it drives the system across an energy threshold. This external input primarily serves to overcome the activation barriers that inhibit plastic deformation (e.g., lattice friction, dislocation pinning).

During the stress reduction process, the relaxation mechanism operates across multiple scales:

Macroscopic scale: Through deformation coordination and geometric stabilization.

Mesoscopic scale: Via the evolution of grain boundaries and the stabilization of precipitated phases.

Microscopic scale: Through dislocation slip/annihilation, magnetic domain wall motion, and grain reorientation.

Essentially, stress relief relies on micro- or macro-plastic deformation to release stored elastic strain energy. Reducing residual stress has a significantly beneficial impact on material properties, including enhanced fatigue life, dimensional stability, and hardness. Therefore, the development and optimization of reduction technologies are of great engineering significance.

Residual stress is a self-equilibrated internal load that is intrinsically linked to the material’s structural geometry and processing history. Typically, residual stress fields are complex and multidimensional; a self-equilibrated stress state generally requires spatial gradients (2D or 3D) rather than simple uniform loading conditions. Common examples include the complex 3D stress fields resulting from welding and the 2D planar stress associated with rolled strip steel.

Technological advancements have facilitated the emergence of diverse reduction technologies beyond traditional methods. Currently, dozens of techniques are available, each tailored to specific materials, structural geometries, and application requirements. Since residual stress distribution is geometry-dependent, the stress profiles in components of varying shapes differ considerably. Consequently, in practical production, it is imperative to select the most appropriate control method based on the specific structure and material characteristics to ensure optimal performance and service life. Emerging technologies (such as magnetic and ultrasonic treatments) generally offer superior efficiency, energy savings, and environmental friendliness compared to their traditional counterparts.

## 5. Emerging Trends: Multi-Field Coupling Technologies

### 5.1. Combined Magnetic–Vibration Stress Relief

For reductions in residual stress for ferromagnetic materials, such as steel strips, magnetic and vibration technologies offer distinct advantages. Magnetic methods can effectively reduce residual stress without physical contact, thereby preserving surface quality, while vibration technology allows for flexible, targeted stress reduction in areas with complex geometries or shape defects. As heterogeneous layered materials and 3D printing become increasingly pivotal in manufacturing, effective residual stress reduction at the heterogeneous interface layer represents a promising direction for future development. By adjusting the frequency and intensity of the electromagnetic field to control penetration depth, coupled with mechanical vibration, residual stress at these critical interfaces can be effectively managed.

**Figure 14:**
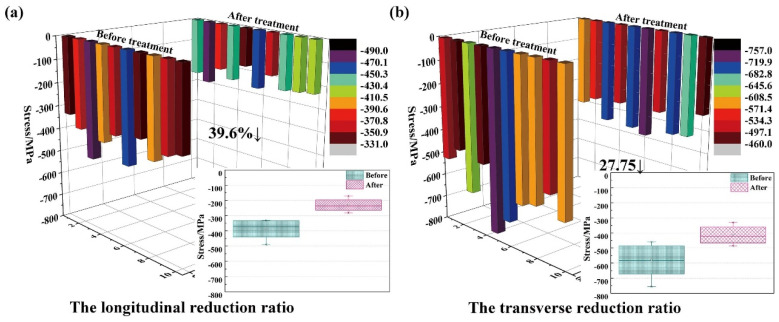
Results of transverse and longitudinal stress reduction before and after the combined magnetic–vibration treatment [110]: (**a**) longitudinal; (**b**) transverse.

Based on the distinct benefits of individual treatments, a novel hybrid approach—Combined Magnetic–Vibration Stress Relief (CMVSR)—has been proposed [106,107,108]. Experimental evidence indicates that this method is not only applicable to large-scale and heavy-section ferromagnetic components but also possesses broad practical implications. Crucially, the transition toward multi-field coupling to combat complex residual stresses is becoming a global consensus in the field. For instance, recent breakthroughs by Li et al. [109] successfully established a comprehensive thermal–metallurgical–mechanical (TMM) model to analyze the laser welding of high-strength steels. Their investigation quantified how martensitic phase transformation and associated volumetric expansion effectively counteract peak tensile stresses during cooling. Parallel to these global advancements, specialized investigations into Combined Magnetic–Vibration Stress Relief (CMVSR) by Huang et al. [110] have identified a robust magneto-mechanical coupling effect. Their study established a dynamic coupled numerical model demonstrating that superimposed alternating magnetic fields significantly lower the mechanical activation energy required for dislocation slip. This synergy enhances stress relief efficacy far beyond the simple superposition of the two individual methods [111,112,113], leading to substantial multi-directional stress reduction (Figure 14).

The underlying mechanisms of this coupling have also been elucidated. Research reveals that the efficacy of vibration treatment depends not only on excitation parameters but also on the component’s geometric dimensions and mode shapes. Similarly, the mechanism of magnetic treatment is highly sensitive to the material’s magnetic domain structure and magnetostrictive properties. It is posited that the dynamic magnetic field induces micro-fatigue or cyclic softening in local high-stress regions, facilitating stress relaxation even at low frequencies and field intensities. This understanding helps define the range of materials most suitable for magnetic-based interventions.

### 5.2. Thermally Assisted Multi-Field Coupling: Thermal–Vibration and Thermal–Magnetic Treatments

While single-field dynamic treatments (such as VSR or magnetic treatment) effectively mitigate macroscopic stresses, their efficacy is occasionally limited by high intrinsic lattice friction or strong dislocation pinning in certain complex alloys. To address this, recent research has pivoted towards thermally assisted multi-field coupling, utilizing low to moderate thermal activation to lower the energy barriers for subsequent dynamic stress relaxation.

**Figure 15:**
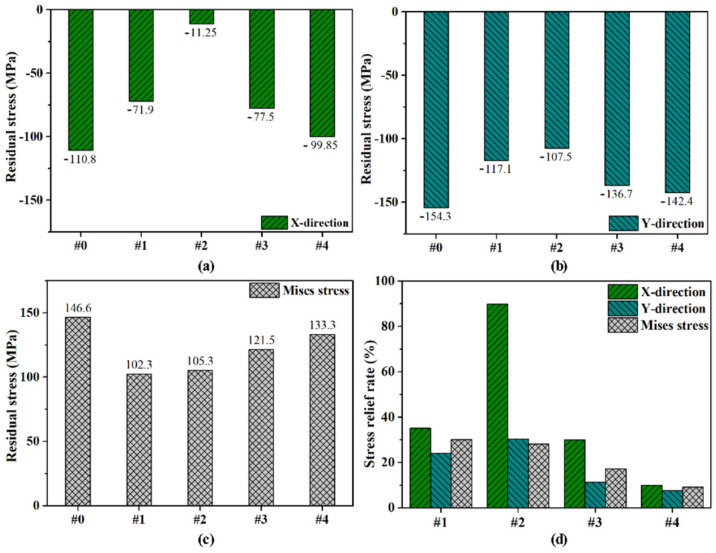
Presentation of the residual stress outcomes in the SiC/Al composites, both pre- and post-stress relief treatments, including the following: (**a**) X-direction stress, (**b**) Y-direction stress, (**c**) von Mises stress, and (**d**) stress relief rate [117].

Thermal–Vibration Stress Relief (TVSR): For paramagnetic or complex non-ferromagnetic alloys, combining sub-recrystallization heating with mechanical vibration has proven highly synergistic. For instance, recent studies on aluminum alloys [114,115] demonstrated that TVSR accelerates the evolution of unstable precipitates and enhances dislocation mobility. The moderate thermal energy weakens the atomic bonding resistance, allowing the superimposed dynamic vibrational stress to initiate plastic micro-yielding much more efficiently than VSR at room temperature, all while avoiding the severe geometric distortion associated with conventional high-temperature annealing [116] (as demonstrated in Figure 15).

Thermal–Magnetic Stress Relief (TMSR): Building on similar thermodynamic principles, for functional ferromagnetic materials (e.g., iron-core silicon steel sheets), Combined Thermal–Magnetic Stress Relief (TMSR) has emerged as a highly validated, eco-friendly alternative. Conventional thermal annealing of silicon steel, while effective for stress relief, often risks altering the optimized magnetic domain structures and incurs massive energy consumption.

**Figure 16:**
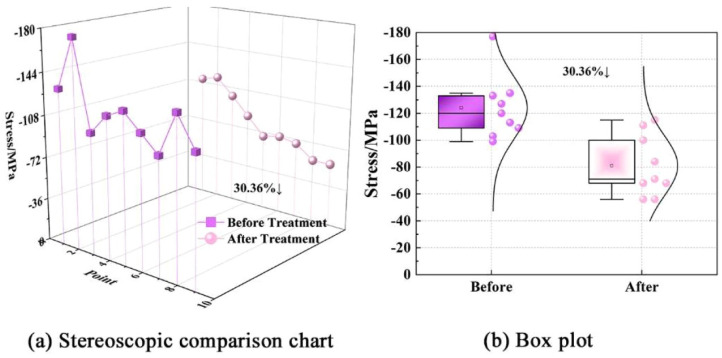
Residual stress changes: (**a**) stereoscopic comparison chart; (**b**) box plot [121].

Recent experimental investigations [118,119,120] have quantitatively validated the TMSR approach. By preheating the material to a moderate temperature well below the recrystallization threshold (100–300 °C), the thermal activation provides sufficient basal energy to mobilize pinned domain walls. When an alternating magnetic field is simultaneously applied, the magnetostrictive effect induces micro-plasticity at stress concentration zones. Quantitative results demonstrate that TMSR can achieve a residual stress reduction efficiency of up to 45–75%, significantly surpassing the linear sum of individual thermal or magnetic treatments (Figure 16). Moreover, because the peak temperature is drastically lower than conventional annealing, this method preserves the material’s critical electromagnetic properties and geometric flatness, positioning TMSR as a highly energy-efficient and industrially viable technology.

### 5.3. Quantitative Comparison of Reduction Methods

Table 1 summarizes the characteristics, mechanisms, and limitations of the various residual stress reduction technologies discussed. To effectively manage residual stresses post-manufacturing, particularly in two-dimensional structures with strict surface quality requirements like iron-core silicon steel sheets, non-contact and highly efficient relaxation strategies are imperative. As indicated in the table, while conventional methods are universally applicable, they often suffer from high energy consumption or geometric distortion. Consequently, the development of multi-field coupling strategies has become a critical research focus to achieve eco-friendly, non-destructive, and highly efficient stress mitigation.

### 5.4. Practical Implementation and Limitations

To translate these multi-field hybrid frameworks into heavy industry applications, critical challenges remain. Regarding energy sustainability, preliminary models suggest that while TMSR requires simultaneous power inputs for electromagnetic excitation and induction heating, the total energy consumption (often measured in kilowatt-hours, kWh) is significantly lower than conventional furnace annealing. For instance, replacing prolonged furnace heating with targeted TMSR can reduce the required kWh per cycle by orders of magnitude due to drastically reduced processing times and low preheating thresholds. However, calibrating excitation parameters for complex, non-uniform geometries necessitates advanced closed-loop control systems. Furthermore, a comprehensive cost–benefit analysis must be considered for industrial scale-up. While the initial capital investment in high-precision multi-field coupling equipment (e.g., dynamic magnetic generators) may be higher than that of conventional annealing furnaces, the significantly lower long-term operating costs and the elimination of massive furnace maintenance make these methods highly cost-effective for precision manufacturing. Another critical hurdle is the current lack of universal international standards (such as ISO or ASTM) governing the application and quality control of emerging multi-field technologies, unlike traditional methods which are well-standardized. Finally, while dynamic treatments effectively relax macroscopic stresses, future research must incorporate long-term fatigue data to verify if the stress relief achieved by these non-thermal hybrid methods provides the same permanent microstructural stability against stress “recovery” as traditional recrystallization annealing.

## 6. Conclusions

(1) Fundamental Impacts and Mechanisms: Residual stress is a complex, self-equilibrating internal load existing across macroscopic, mesoscopic, and microscopic scales. While compressive residual stresses can sometimes be engineered to enhance fatigue life, deleterious tensile residual stresses fundamentally compromise material strength, formability, and long-term dimensional stability. A rigorous understanding of these multi-scale mechanisms is the prerequisite for implementing precise stress mitigation.
(2) Limitations of Current Technologies: A critical evaluation of existing single-field reduction methods reveals inherent trade-offs. Traditional thermal annealing provides effective relaxation but is fundamentally limited by high energy consumption, environmental pollution, and the risk of microstructural degradation. Conversely, mechanical approaches (e.g., tensioning, hammering, and explosion) and vibration stress relief (VSR) suffer from geometric constraints, noise pollution, and high distortion risks. While emerging non-thermal techniques (ultrasonic, magnetic, electropulsing) offer cleaner alternatives, their efficacy is often restricted by material specificities (e.g., ferromagnetism, electrical conductivity) or limited penetration depths.
(3) The Paradigm Shift to Multi-Field Coupling: To overcome the bottlenecks of single-field treatments, the integration of multi-field coupling represents the definitive future of stress relief in modern machinery manufacturing. Novel hybrid techniques—specifically Combined Magnetic–Vibration (CMVSR), Thermal–Vibration (TVSR), and Thermal–Magnetic (TMSR) stress relief—demonstrate profound synergistic effects. By utilizing moderate thermal or magnetic activation to lower the mechanical energy barriers for dislocation slip and domain wall motion, these coupled strategies achieve superior stress homogenization. They offer a highly efficient, energy-saving, and non-destructive solution, establishing a robust foundation for the next generation of eco-friendly advanced manufacturing.

## Figures and Tables

**Table 1:** Characteristics and comparison of residual stress reduction technologies.

	Energy Source	Operational Mode	Typical Working Parameters & Ranges	Target Application	Efficacy & Limitations
Natural Aging	Natural Thermal	Non-contact, Continuous	Ambient temperature; Time: 6–24 months	Castings, forgings, weldments	5–20% stress reduction
Thermal Annealing	Artificial thermal	Non-contact, Continuous	Peak Temp: 500–700 °C; Hold time: 2–10 h	Universally applicable	40–80% stress reduction; High energy cost
Mechanical Tensioning	Mechanical	Contact, Cyclic		Plastically deformable weldments	10–30% stress reduction; High distortion risk
Hammer Peening	Mechanical	Contact, Cyclic		Localized weld zones	20–40% localized reduction; Distortion risk
Explosion	Artificial thermal	Contact, Pulsed		Heavy-duty macro-structures	30–70% stress reduction; Severe safety/distortion risks
Vibration (VSR)	Mechanical	Contact, Cyclic	Frequency: 20–100 Hz; Time: 15–45 min	Universally applicable	20–40% stress reduction; Geometry-dependent
Magnetic (MSR)	Magnetic	Non-contact, Cyclic	Field Intensity: 0.5–2.0 T; Frequency: 10–50 Hz	Ferromagnetic materials	10–40% stress reduction; Material-specific
Electropulsing (EPT)	Electrical	Contact, Pulsed	Current Density: 10^3^–10^4^ A/mm^2^; Duration: <1 min	Conductive metals	40–60% stress reduction; High operational cost
Magnetic–Vibration	Mechanical + Magnetic	Mixed, Cyclic	Field: 1.0–2.0 T + Frequency: 20–100 Hz;	Universally applicable	30–60% stress reduction; Low cost, Distortion risk
Thermal–Vibration	Thermal + Mechanical	Mixed, Continuous/Cyclic	Temp: 100–300 °C + Frequency: 20–100 Hz;	Universally applicable	30–65% stress reduction; Moderate cost/distortion
Thermal–Magnetic	Thermal + Magnetic	Non-contact, Mixed	Temp: 100–300 °C + Field: 1.0–2.0 T	Universally applicable	40–75% stress reduction; Low cost, No distortion

## Data Availability

No new data were created or analyzed in this study. Data sharing is not applicable to this article.
